# The positive clinical therapeutically effects of Escin on advanced thyroid cancer

**DOI:** 10.1002/cam4.1031

**Published:** 2017-04-04

**Authors:** Jin‐Yu Mei, Ming‐Jun Zhang, Yuan‐Yuan Wang, Ye‐Hai Liu

**Affiliations:** ^1^ Department of OtolaryngologyThe First Hospital of Anhui Medical UniversityHefei230031China; ^2^ Department of OtolaryngologyThe Second Hospital of Anhui Medical UniversityHefei230601China; ^3^ Department of OncologyThe Second Hospital of Anhui Medical UniversityHefei230601China; ^4^ Department of PharmacyThe Second Hospital of Anhui Medical UniversityHefei230601China

**Keywords:** Advanced thyroid cancer, Escin, pharmacokinetics, side effects

## Abstract

The incidences of thyroid cancer keep rising worldwide over the past few decades. Although most thyroid cancers are indolent and highly curable, the treatment for advanced thyroid cancer remains challengeable in clinical practice. We performed two separate cohorts to evaluate the safety and efficiency of Escin in patients with advanced thyroid cancer . In cohort 1, 120 patients were divided into four groups equally and were administrated with placebo or different dosages of Escin. The pharmacokinetics of Escin and the side effects were evaluated. In cohort 2, 120 patients were treated with Escin. Several biomarkers related to the progression of thyroid cancer were evaluated. Kaplan–Meier (KM) analyses were performed to evaluate progression‐free survival (PFS) and overall survival (OS). The serum Escin concentrations were stable during the treatment. Escin (0.6 mg/kg/day for 9 days, intravenous injection) was tolerable for patients with thyroid cancer . Escin significantly reduced the serum levels of TSH, TgAb, Tg, and calcitonin and prolonged the PFS and OS for patients with advanced thyroid cancer. This study showed Escin is efficient and well tolerated in patients with advanced thyroid cancer. Future studies are needed to investigate the mechanism of Escin on thyroid cancer and the proper dosage of Escin clinically.

## Introduction

The incidences of thyroid cancer keep rising worldwide over the past few decades. Nowadays, thyroid cancer is the eighth and the fifth most common cancer in women in the China 1 and USA 2, respectively. An estimated around 67,778 and 62,000 new female cases occurred in China 1 and USA 2 annually. In China, this rapid increased incidence of thyroid cancer might owe to the development of screening techniques and iodine salt supplementation 3. The first choice of treatments in nearly all patients with thyroid cancer is surgery, with most patients undergoing total (86%) or partial (12%) thyroidectomy 4. The following options of treatments are radioactive iodine (I‐131) for papillary or follicular thyroid cancer 5 and radiation therapy for medullary thyroid cancer. The prognosis of thyroid cancer is good among all types of cancer. A recent statistical analysis showed the overall 5‐year survival rate for patients with thyroid cancer who were diagnosed during 2005 through 2011 is 98% 4. Although most thyroid cancers are indolent and highly curable, the treatment for advanced thyroid cancer remains challengeable in clinical practice.

Many studies have shown multiply oncogenic pathways are involved with the tumor genesis, therapy resistance, and recurrence of thyroid cancer, such as the MAPK signaling pathway 6, the PI3K‐AKT signaling pathway 7, the NF‐*κ*B signaling pathway 8, the WNT‐*β*‐catenin signaling pathway 9, and the thyroid‐stimulating hormone receptor signaling pathway 10. Utilizing the kinase inhibitors related to these signaling pathways for advanced thyroid cancer was carefully examined in the past few years. FDA has already approved many kinase inhibitors for advanced thyroid cancer treatment, such as sorafenib 11 and lenvatinib 12 for advanced differentiated thyroid cancer; vandetanib 13 and cabozantinib 14 for advanced medullary thyroid cancer; and vemurafenib 15 and lenvatinib 16 for anaplastic thyroid cancer. Although many targeted therapy drugs are developed and applied for advanced thyroid cancer treatment, the 5‐year relative survival rates of anaplastic thyroid cancer most of whom are advanced stage are low to 9% 4. Thus, more agents for advanced thyroid cancer treatment are still needed to be investigated.

Escin is a natural mixture of pentacyclic triterpenoid which is isolated from the seeds of Aesculus hippocastanum 17. Escin has shown multiply pharmacological effects including anti‐inflammatory, vascular protection, and a venotonic effect 18. In traditional Chinese medicine, Escin is widely used for the treatment of cerebral edema, and chronic venous insufficiency, among other conditions 19, while recent studies showed Escin might have antitumor effects as well. Ciftci and his colleagues 20 found Escin reduced cell proliferation and induces apoptosis on glioma and lung adenocarcinoma cell lines; Wang et al. 21 found Escin suppressed the metastasis of triple‐negative breast cancer cells through inhibiting epithelial–mesenchymal transition. Furthermore, study also showed Escin can reverse multidrug resistance through inhibition of the GSK3*β*/*β*–catenin pathway in cholangiocarcinoma cells 22. Clinically, Escin has been proved to be well tolerated and efficiency on improving the gastrointestinal motility in patients with colorectal cancer as well 19.

As Escin showed significant antitumor effects in many types of cancer cells and is well tolerated in patients with colorectal cancer, we hypothesize that Escin may have some effects on patients with advance thyroid cancer. Here, we conducted two cohorts of clinical studies to investigate the safety and efficiency of Escin on patients with advanced thyroid cancer.

## Methods

### Patients and healthy volunteers

Eligible patients of our studies were ≥18 years old with histologically confirmed advanced thyroid carcinoma (including papillary, follicular, Hürthle cell, and poorly differentiated). All patients have already received standard surgery with or without radioactive iodine treatment or radiation therapy following NCCN guideline. The Eastern Cooperative Oncology Group performance statuses of our patients are between 0 and 2. The pervious chemotherapy or biologic treatment (kinase inhibitor or vaccine) was allowed but not within a month of treatment. The exclusion criteria included significant cardiac, hematopoietic, hepatic, or renal dysfunction. Hundred healthy volunteers were recruited from The First Hospital of Anhui Medical University. All the volunteers were 18–50 years old and have no history or evidence of clinically chronic systemic disease. Pregnant or lactating women were excluded as well. All patients and volunteers were needed to sign the consent forms, and this study was approved by medical ethics committee of The First Hospital of Anhui Medical University.

### Study design

We performed two separate cohorts to evaluate the safety and efficiency of Escin in patients with advanced thyroid cancer. In cohort 1, 120 patients were divided into four groups equally; patients in each group were administrated with placebo, Escin 0.2 mg/kg/day (Shandong Luye Pharmaceutical Company, China), 0.4 mg/kg/day, or 0.6 mg/kg/day, respectively, for 9 days. Escin was diluted in 5% dextrose. One hour right after the Escin injection, the blood samples were taken from the patients and Escin concentrations in plasma were tested. Treatment‐related adverse effects (AE) and laboratory values were recorded and assessed according to the Common Terminology Criteria for Adverse Events, version 3.0 in all the patients as well.

In cohort 2, 120 patients were treated with Escin 0.6 mg/kg/day for 9 days. Thyroid function of all the patients (such as TSH, TBG, and TgAb) was evaluated before and after the Escin treatment and compared with that of 100 healthy volunteers. The recurrence time and death time of each patient were recorded, and the Kaplan–Meier (KM) analysis was performed to compare the progression‐free survival (PFS) and overall survival (OS) between the 120 patients from cohort 2 and 30 patients from cohort 1 who received placebo treatment.

### Evaluations and assessments

Serum thyroid‐stimulating hormone (TSH), total or free triiodothyronine (T3), total or free thyroxine (T4), thyroglobulin (Tg), antithyroglobulin antibody (TgAb), thyroid binding globulin (TBG), and calcitonin levels are measured before and after Escin treatment in patients with cancer from cohort 2 and healthy volunteers. Standard computed tomography/magnetic resonance imaging of neck for tumor assessment was performed at baseline, approximately every 3 month after Escin treatment. Scans of other organs for metastasis were performed only when clinically indicated.

### Statistical analysis

Results are presented as mean ± SD. Student's *t*‐test was used to compare the results related to thyroid function between the patients with thyroid cancer and healthy volunteers. KM analysis was used to compare the PFS and OS between patients with or without Escin treatment. All the analyses were performed according to the intention‐to‐treat principle. The commercial SPSS (version 16.0, SPSS Inc, Chicago, IL) for Windows was used for all the analysis. *P* < 0.05 were considered as statistically significant.

## Results

### Patients’ characteristics

We performed two separate cohorts to evaluate the safety and efficiency of Escin in patients with advanced thyroid cancer. Between September 2013 and October 2015, 240 patients with advanced thyroid cancer and 100 healthy volunteers were recruited from The First Hospital of Anhui Medical University. In the first part of the study, 120 patients were enrolled and received Escin 0.2 mg/kg/day (intravenous injection, i.v.) (cohort 1; *n* = 30), Escin 0.4 mg/kg/day i.v. (cohort 1; *n* = 30), Escin 0.6 mg/kg/day i.v. (cohort 1; *n* = 30), or placebo (cohort 1; *n* = 30) for 9 days. In cohort 2, 120 patients were treated with Escin 0.6 mg/kg/day i.v. (cohort 2; *n* = 120) for 9 days. The characteristics of the 240 patients and 100 healthy volunteers are shown in Table 1. As shown in Table 1, there are no differences among the patients from different Escin dosage groups of cohort 1, and there is no difference between the patients from cohort 1 and cohort 2. Although the age and sex fraction are the same between the healthy volunteers and patients with cancer, most of patients had the symptoms of diarrhea, dyspnea, dysphagia, or corestenoma which related to pervious cancer treatments, while no healthy volunteers showed these kinds of symptoms.

**Table 1:** Patient characteristics

Variable	First study of patient (*n* = 120)	Second study of patient (*n* = 120)	Control (*n* = 100)	Significant difference (1^st^ vs 2^nd^ study; Yes/No)	Significant difference (1^st^/2^nd^ study vs control; Yes/No)
Age: year	69.6 ± 4.3	64.8 ± 4.9	68.2 ± 2.3	No	No
Sex: no. (%)					
Male	72 (60)	66 (55)	50 (50)	No	No
Female	48 (40)	54 (45)	50 (50)	No	No
Hyperlipidemia (*n*, %)	108 (90)	102 (85)	0 (0)	No	Yes
Coronary artery disease (*n*, %)	96 (80)	108 (90)	0 (0)	No	Yes
Diabetes mellitus (*n*, %)	120 (100)	120 (100)	0 (0)	No	Yes
Atrial fibrillation (*n*, %)	108 (90)	114 (95)	0 (0)	No	Yes
Palpitation (*n*, %)	120 (100)	120 (100)	0 (0)	No	Yes
Face flushing (*n*, %)	108 (90)	114 (100)	0 (0)	No	Yes
Diarrhea (*n*, %)	114 (95)	120 (100)	3 (3)	No	Yes
Hoarseness (*n*, %)	120 (100)	114 (95)	0 (0)	No	Yes
Bucking (*n*, %)	102 (85)	102 (85)	0 (0)	No	Yes
Dyspnea (*n*, %)	108 (90)	102 (85)	0 (0)	No	Yes
Dysphagia (*n*, %)	120 (100)	120 (100)	0 (0)	No	Yes
Lymphadenectasis (*n*, %)	120 (100)	120 (100)	0 (0)	No	Yes
Corestenoma (*n*, %)	120 (100)	120 (100)	0 (0)	No	Yes

Student's *t*‐test was used to compare the results.

### Pharmacokinetics of Escin

**Figure 1:**
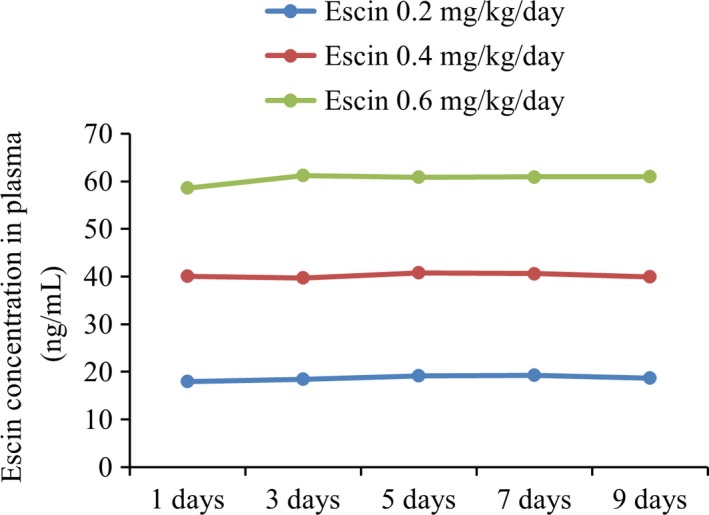
Escin had stable blood concentration during cohort 1. Mean Escin plasma concentration vs. time profiles from patients dosed with Escin 0.2 mg/kg/day (*n* = 30), Escin 0.4 mg/kg/day (*n* = 30), and 0.6 mg/kg/day (*n* = 30). Each value represents the mean ± SD.

To investigate the pharmacokinetics of Escin, blood samples were taken one hour after Escin treatment from the patients of different Escin dosage treatment groups of cohort 1. Serum Escin concentrations were determined from each samples. As shown in Figure 1, the serum Escin concentrations are quite stable among each group during the treatment. The average of Escin serum concentration was around 20 ng/mL in the 0.2 mg/kg/day group (*n* = 30), 40 ng/mL in 0.4 mg/kg/day group (*n* = 30), and 60 ng/mL in 0.6 mg/kg/day group (*n* = 30), suggesting a dose‐dependent increase in Escin serum concentration.

### Safety and tolerability

To investigate the tolerability of Escin, common adverse events (any grade 3 or higher) from the cohort 1 are summarized in Table 2. There are no adverse events reported in 0.2 mg/kg/day and 0.4 mg/kg/day groups; while in Escin 0.6 mg/kg/day group, one patient (3%) occurred grade 3 Anemia, another patient (3%) occurred grade 3 ALT elevations. After symptomatic treatment, these two patients accomplished 9‐day Escin treatment.

**Table 2:** All adverse a dose‐limiting toxicity (DLT) during first study

	30 patients with 0.2 mg/kg Escin. No. (%)	30 patients with 0.4 mg/kg Escin. No. (%)	30 patients with 0.6 mg/kg Escin. No. (%)
Hyperbilirubinemia	0 (0)	0 (0)	0 (0)
AST elevation	0 (0)	0 (0)	0 (0)
Anemia	0 (0)	0 (0)	1 (3)
Thrombocytopenia	0 (0)	0 (0)	0 (0)
Fatigue	0 (0)	0 (0)	0 (0)
Neutropenia	0 (0)	0 (0)	0 (0)
Rash	0 (0)	0 (0)	0 (0)
Diarrhea	0 (0)	0 (0)	0 (0)
Fever	0 (0)	0 (0)	0 (0)
Bleeding	0 (0)	0 (0)	0 (0)
Mucositis	0 (0)	0 (0)	0 (0)
Hypocalcemia	0 (0)	0 (0)	0 (0)
ALT elevation	0 (0)	0 (0)	1 (3)
Hyponatremia	0 (0)	0 (0)	0 (0)
Hypophosphatemia	0 (0)	0 (0)	0 (0)
Sudden death	0 (0)	0 (0)	0 (0)

Unless otherwise noted, all data presented as *n* (%).

### Efficacy

In cohort 2, we tried to investigate whether Escin has therapeutic effects on patients with advanced thyroid cancer. Firstly, we measured several indexes of thyroid function before and after Escin treatment to see whether Escin can improve thyroid function in patients with cancer. Indeed, as shown in Table 3, after 9‐day Escin treatments TSH level dropped from 1.87 ± 0.64 IU/L to 1.1 ± 0.34 IU/L, TgAb level dropped from 54.9 ± 8.14 IU/mL to 44.77 ± 4.28 IU/mL, TBG level dropped from 78.19 ± 7.23 *μ*g/L to 54.1 ± 6.87 *μ*g/L, and calcitonin level dropped from 198.32 ± 35.98 ng/L to 124.32 ± 28.32 ng/L, respectively, suggesting Escin treatment might regulate the thyroid function in patients with thyroid cancer. As TSH, TgAb, Tg, and calcitonin increasing are well‐known indicators for thyroid cancer recurrence and Escin is shown to be able to decrease these indexes simultaneously, it became very interesting to see whether the effects Escin on these indicators would lead to benefits on long‐term survive rate of patients with advanced thyroid cancer.

**Table 3:** All significant adverse events for thyroid cancer during second study (*P* < 0.5)

Variable	Second study of patient *N* = 120	Second study of patient with treatment *N* = 120	Control *N* = 100	Significant difference between second study and control (*P* < 0.5)
TSH (IU/L)	1.1 ± 0.34	1.87 ± 0.64	2.34 ± 0.76	*P* < 0.5
Total T3 (ng/mL)	5.81 ± 1.23	3.21 ± 0.77	1.27 ± 0.25	*P* < 0.5
Total T4 (*μ*g/dL)	22.68 ± 3.24	2.64 ± 1.08	10.1 ± 2.09	*P* < 0.5
Free T3 (pg/mL)	5.6 ± 1.41	4.35 ± 1.07	3.21 ± 1.35	*P* < 0.5
Free T4 (ng/dL)	2.61 ± 0.13	1.60 ± 0.11	1.34 ± 0.22	*P* < 0.5
TG (ng/mL)	55.98 ± 7.41	25.78 ± 2.14	10.29 ± 2.66	*P* < 0.5
TGA(IU/mL)	54.9 ± 8.14	44.77 ± 4.28	22.57 ± 4.92	*P* < 0.5
TBG(*μ*g/L)	78.19 ± 7.23	54.1 ± 6.87	35.44 ± 4.17	*P* < 0.5
Calcitonin (ng/L)	198.32 ± 35.98	124.32 ± 28.32	89.67 ± 11.44	*P* < 0.5

Unless otherwise noted, all data presented as *n* (%).

IQR, interquartile range; TSH, thyroid‐stimulating hormone; T3, triiodothyronine; T4, thyroxine.

**Figure 2:**
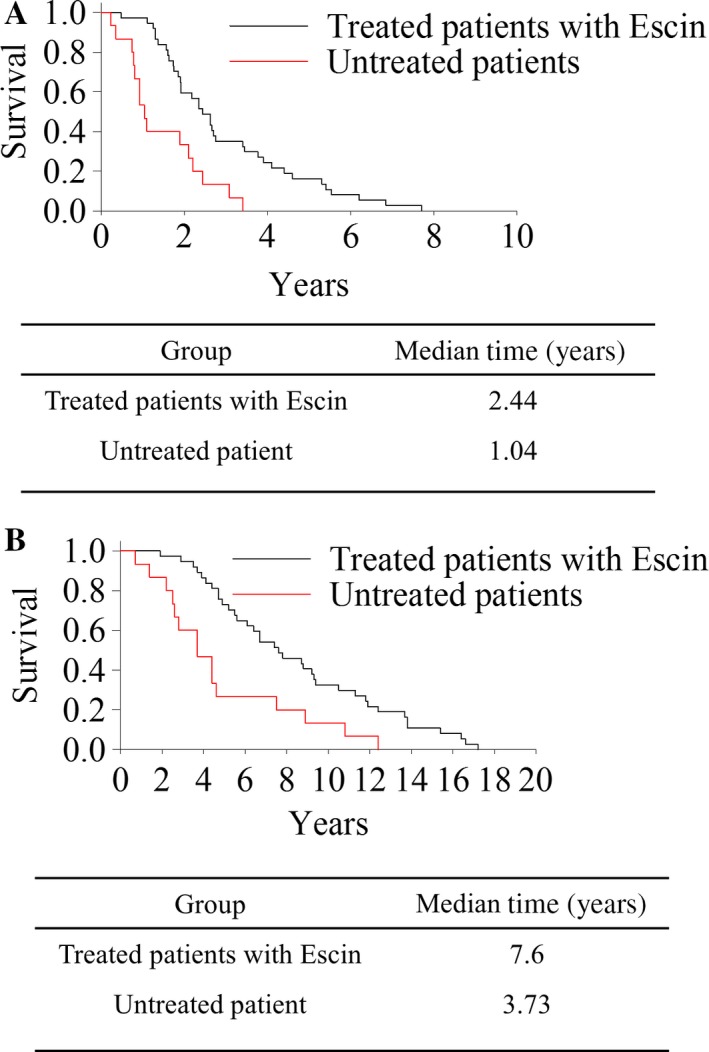
Escin inhibited the recurrence of cancer and prolonged the survival time of patients. (A) The recurrence time was recorded of 120 patients of second study compared 30 patients of first study without Escin treatment as control from surgery and radiation therapy (*P* < 0.01). (B) The survival time was recorded of 120 patients of second study compared 30 patients of first study without Escin treatment as control from surgery and radiation therapy (*P* < 0.01).

We then evaluated the response rate of Escin treatment as shown in Table 4. Among the 120 patients in cohort 2, 12 (10%) had a partial response, 90 (75%) had stable disease, 18 (15%) had progressive disease, and the disease control rate was 102 (85%). After low term follow‐up, we compared the progression‐free survival (PFS) and overall survival (OS) between the 120 patients from cohort 2 who have received the Escin treatment and the 30 patients from cohort 1 who have received placebo. As shown in Figure 2, significant prolongation of PFS and OS was observed for patients receiving Escin compared with placebo. The median PFS was 2.44 years in the Escin‐treated group comparing with 1.04 years in placebo group. The median OS was also significantly improved from 3.73 years (placebo group) to 7.6 years (Escin‐treated group).

**Table 4:** Response rates using the response evaluation criteria in patients in second study

Response	Number of patients (%)
Complete response	0 (0)
Partial response	12 (10)
Stable disease	90 (75)
Progression disease	18 (15)
Disease control rate (DCR)	102 (85)

Unless otherwise noted, all data presented as *n* (%).The disease control rate was defined as the proportion of patients who had a best response rating of a complete response, partial response, or stable disease that was maintained for ≥4 weeks from the first manifestation of the rating.

## Discussion

In our current study treating advanced thyroid cancer patients with Escin, we found Escin was well tolerated and significantly improved the progression‐free survival and overall survival of patients with advanced thyroid cancer.

To monitor the thyroid cancer progression and recurrence, clinicians test the levels of several biomarkers regularly as the indicators, such as TSH, TgAb, Tg, and calcitonin. Circulating TSH is shown to stimulate proliferation in most thyroid cancer cells 23. Pervious study showed reducing TSH concentrations by the administration of L‐T4 after routine treatments can significantly improve clinical outcomes in patients with advanced thyroid cancer 24, 25. The trends of serum TgAb level can reflect changes of thyroid tissue mass; thus, serum TgAb concentrations also serve as a surrogate postoperative differentiated thyroid cancer tumor marker clinically. A rising level of TgAb suggests cancer recurrence, whereas a progressive decline suggests successful treatment 26. Thyroglobulin (Tg) is another important biomarker for monitoring the progression of thyroid cancer especially papillary thyroid cancer. Achieving an undetectable serum Tg level after surgical and radioiodine treatment is associated with low recurrence rates and has been termed biochemical remission 27. Calcitonin secreted by the thyroid C cells which give rise of the medullary thyroid carcinoma (MCT) is a sensitive biomarker for diagnosis and follow‐up in medullary thyroid cancer (MTC) especially for decades 28. In our current study, all the patients showed higher serum level of TSH, TgAb, Tg, and calcitonin after surgery and radiation therapy as shown in Table 3, while 9 days of Escin treatment significantly reduced the serum levels of all these four biomarkers close to the normal level of healthy volunteers suggesting Escin might benefit all subtypes of thyroid cancer.

In traditional Chinese medicine, Escin has been widely used to promote appetite, prevent gas formation of gastrointestinal tract, and documented as an antiseptic, anti‐edematous, and anti‐inflammatory agent in various disease models 29. Studies have shown the major mechanisms for the anti‐inflammatory effects of Escin are through modulation of NF‐*κ*B signaling pathway 29. As chronic inflammation and the incidence or the recurrence of thyroid cancer are highly related 30 and, moreover, increased NF‐*κ*B activation in thyroid cancer cell lines and tissues has long been documented 8, we hypothesize that Escin would benefit the patients with thyroid cancer. Furthermore, many recent studies showed Escin might have antitumor effects in various cancer types. Thus, we conducted current clinical study to investigate the antitumor effect of Escin on patients with advanced thyroid cancer.

To our knowledge, this is the first clinic study of Escin with thyroid cancer patients. Escin showed striking effects on prolonging the progression‐free survival and overall survival in our current patients. This kind of antitumor effect is consistent with other in vitro and in vivo nude mice studies showing Escin has antitumor effects on lung cancer 20, breast cancer 21, liver cancer 31, pancreas cancer 32, and colon cancer 33. The dosage of Escin in our current was 0.6 mg/kg/day i.v. for 9 days. In our study, there are only two patients (6%) developed grade 3 side effects, one occurred Anemia, another showed ALT elevations. While after symptomatic treatment, both patients accomplished 9‐day Escin treatment. Our dosage of Escin is higher than another clinical study investigating whether Escin can improve gastrointestinal motility in patients with colon cancer after surgery 19. In that study, the highest dose of Escin was 25 mg/day i.v for 7 days. There are no severe side effects reported in that trial either. Since at current dosage, Escin is well tolerated and shows some promising antitumor effects on patients with cancer. Further studies need to investigate the feasibility of higher dose or longer duration of Escin usage.

Escin showed different antitumor mechanisms in various types of cancer. For example, in pancreas cancer 32, Wang and his colleague found Escin potentiated the antitumor activity of gemcitabine via the inactivation of NF‐*κ*B. While in cholangiocarcinoma 22, Escin was found to reverse P‐gp‐dependent multidrug resistance via inhibition of the GSK3*β*/*β*–catenin pathway. Because Escin has different antitumor mechanisms according to different cancer types, understanding via which pathway Escin affects the thyroid cancer cells become interesting and necessary. Better understanding of the mechanism of Escin on thyroid cancer will help us to find some biomarkers to identify the most suitable subgroup of patients with thyroid cancer for Escin treatment.

In conclusion, our current study showed Escin is efficient and well tolerated in patients with advanced thyroid cancer. Based on the safety, and pharmacokinetic and efficacy profiles of our study, it was concluded that Escin 0.6 mg/kg/day i.v. for 9 days was tolerable. Furthermore, Escin significantly reduced the serum levels of TSH, TgAb, Tg, and calcitonin related to thyroid cancer progression and recurrence and prolonged the PFS and OS for patients with advanced thyroid cancer. Future studies are needed to investigate the mechanism of Escin on thyroid cancer and the proper dosage of Escin clinically.

## Funding Information

No funding information provided.

## Conflicts of Interest

The authors declare that there is no conflict of interests.

## References

[1] Chen, W. , R. Zheng , H. Zeng , S. Zhang , and J. He . 2015 Annual report on status of cancer in China, 2011. Chin. J. Cancer Res. 27:2–12. 25717220 10.3978/j.issn.1000-9604.2015.01.06 PMC4329176

[2] Cabanillas, M. E. , D. G. McFadden , and C. Durante . 2016 Thyroid cancer. Lancet 388:2783‐2795. 27240885 10.1016/S0140-6736(16)30172-6

[3] Zhou, X. , C. Zhang , M. Wang , L. Yu , and M. Yan . 2015 Dezocine for Preventing Postoperative Pain: A Meta‐Analysis of Randomized Controlled Trials. PLoS ONE 10:e0136091. 26287536 10.1371/journal.pone.0136091 PMC4545891

[4] Miller, K. D. , R. L. Siegel , C. C. Lin , A. B. Mariotto , J. L. Kramer , J. H. Rowland , et al. 2016 Cancer treatment and survivorship statistics, 2016. CA Cancer J, Clin. 10.3322/caac.21349 27253694

[5] Haymart, M. R. , M. Banerjee , A. K. Stewart , R. J. Koenig , J. D. Birkmeyer , and J. J. Griggs . 2011 Use of radioactive iodine for thyroid cancer. JAMA 306:721–728. 21846853 10.1001/jama.2011.1139 PMC3352591

[6] Xing, M. 2008 Recent advances in molecular biology of thyroid cancer and their clinical implications. Otolaryngol. Clin. North Am. 41:1135–1146. 19040974 10.1016/j.otc.2008.07.001 PMC2615411

[7] Saji, M. , and M. D. Ringel . 2010 The PI3K‐Akt‐mTOR pathway in initiation and progression of thyroid tumors. Mol. Cell. Endocrinol. 321:20–28. 19897009 10.1016/j.mce.2009.10.016 PMC2849843

[8] Mitsiades, C. S. , D. McMillin , V. Kotoula , V. Poulaki , C. McMullan , J. Negri , et al. 2006 Antitumor effects of the proteasome inhibitor bortezomib in medullary and anaplastic thyroid carcinoma cells in vitro. J. Clin. Endocrinol. Metabol. 91:4013–4021. 10.1210/jc.2005-2472 16849420

[9] Wiseman, S. M. , O. L. Griffith , A. Gown , B. Walker , and S. J. Jones . 2011 Immunophenotyping of thyroid tumors identifies molecular markers altered during transformation of differentiated into anaplastic carcinoma. Am. J. Surg. 201:580–586. 21545903 10.1016/j.amjsurg.2011.01.010

[10] Kimura, T. , A. Van Keymeulen , J. Golstein , A. Fusco , J. E. Dumont , and P. P. Roger . 2001 Regulation of thyroid cell proliferation by TSH and other factors: a critical evaluation of in vitro models. Endocr. Rev. 22:631–656. 11588145 10.1210/edrv.22.5.0444

[11] Brose, M. S. , C. M. Nutting , B. Jarzab , R. Elisei , S. Siena , L. Bastholt , et al. 2014 Sorafenib in radioactive iodine‐refractory, locally advanced or metastatic differentiated thyroid cancer: a randomised, double‐blind, phase 3 trial. The Lancet 384:319–328. 10.1016/S0140-6736(14)60421-9 PMC4366116 24768112

[12] Schlumberger, M. , M. Tahara , L. J. Wirth , B. Robinson , M. S. Brose , R. Elisei , et al. 2015 Lenvatinib versus placebo in radioiodine‐refractory thyroid cancer. N. Eng. J. Med. 372:621–630. 10.1056/NEJMoa1406470 25671254

[13] Wells, S. A. , B. G. Robinson , R. F. Gagel , H. Dralle , J. A. Fagin , M. Santoro , et al. 2012 Vandetanib in patients with locally advanced or metastatic medullary thyroid cancer: a randomized, double‐blind phase III trial. J. Clin. Oncol. 30:134–141. 22025146 10.1200/JCO.2011.35.5040 PMC3675689

[14] Elisei, R. , M. J. Schlumberger , S. P. Müller , P. Schöffski , M. S. Brose , M. H. Shah , et al. 2013 Cabozantinib in progressive medullary thyroid cancer. J. Clin. Oncol. 31:3639–3646. 24002501 10.1200/JCO.2012.48.4659 PMC4164813

[15] Hyman, D. M. , I. Puzanov , V. Subbiah , J. E. Faris , I. Chau , J.‐Y. Blay , et al. 2015 Vemurafenib in multiple nonmelanoma cancers with BRAF V600 mutations. N. Eng. J. Med. 373:726–736. 10.1056/NEJMoa1502309 PMC4971773 26287849

[16] Takahashi, S. , M. Tahara , and N. Kiyota . 2013 Phase II study of lenvatinib, a multitargeted tyrosine kinase inhibitor, in patients with all histologic subtypes of advanced thyroid cancer (differentiated, medullary, and anaplastic). Ann. Oncol. 24:iv340–iv356

[17] Matsuda, H. , Y. Li , T. Murakami , K. Ninomiya , J. Yamahara , and M. Yoshikawa . 1997 Effects of escins Ia, Ib, IIa, and IIb from horse chestnut, the seeds of Aesculus hippocastanum L., on acute inflammation in animals. Biol. Pharm. Bull. 20:1092–1095. 9353571 10.1248/bpb.20.1092

[18] Guillaume, M. , and F. Padioleau . 1994 Veinotonic effect, vascular protection, antiinflammatory and free radical scavenging properties of horse chestnut extract. Arzneimittelforschung 44:25–35. 8135874

[19] Xie, Q. , X. Zong , B. Ge , S. Wang , J. Ji , Y. Ye , et al. 2009 Pilot postoperative ileus study of escin in cancer patients after colorectal surgery. World J. Surg. 33:348–354. 19052813 10.1007/s00268-008-9816-1

[20] Ciftci, G. A. , A. Iscan , and M. Kutlu . 2015 Escin reduces cell proliferation and induces apoptosis on glioma and lung adenocarcinoma cell lines. Cytotechnology 67:893–904. 25906387 10.1007/s10616-015-9877-6 PMC4545433

[21] Wang, Y. , X. Xu , P. Zhao , B. Tong , Z. Wei , and Y. Dai . 2016 Escin Ia suppresses the metastasis of triple‐negative breast cancer by inhibiting epithelial‐mesenchymal transition via down‐regulating LOXL2 expression. Oncotarget 7:23684–23699. 27008697 10.18632/oncotarget.8152 PMC5029656

[22] Huang, G. L. , D. Y. Shen , C. F. Cai , Q. Y. Zhang , H. Y. Ren , and Q. X. Chen . 2015 beta‐escin reverses multidrug resistance through inhibition of the GSK3beta/beta‐catenin pathway in cholangiocarcinoma. World J. Gastroenterol. 21:1148–1157. 25632187 10.3748/wjg.v21.i4.1148 PMC4306158

[23] Biondi, B. , S. Filetti , and M. Schlumberger . 2005 Thyroid‐hormone therapy and thyroid cancer: a reassessment. Nature Rev. Endocrinol. 1:32–40. 10.1038/ncpendmet0020 16929364

[24] Jonklaas, J. , N. J. Sarlis , D. Litofsky , K. B. Ain , S. T. Bigos , J. D. Brierley , et al. 2006 Outcomes of patients with differentiated thyroid carcinoma following initial therapy. Thyroid 16:1229–1242. 17199433 10.1089/thy.2006.16.1229

[25] Carhill, A. A. , D. R. Litofsky , D. S. Ross , J. Jonklaas , D. S. Cooper , J. D. Brierley , et al. 2015 Long‐term outcomes following therapy in differentiated thyroid carcinoma: NTCTCS registry analysis 1987–2012. J. Clin. Endocrinol. Metab. 100:3270–3279. 26171797 10.1210/JC.2015-1346 PMC5393522

[26] Spencer, C. , and S. Fatemi . 2013 Thyroglobulin antibody (TgAb) methods–Strengths, pitfalls and clinical utility for monitoring TgAb‐positive patients with differentiated thyroid cancer. Best Pract. Res. Clin. Endocrinol. Metab. 27:701–712. 24094640 10.1016/j.beem.2013.07.003

[27] Hughes, D. T. , B. S. Miller , M. S. Cohen , G. M. Doherty , and P. G. Gauger . 2014 Outcomes of total thyroidectomy with therapeutic central and lateral neck dissection with a single dose of radioiodine in the treatment of regionally advanced papillary thyroid cancer and effects on serum thyroglobulin. Ann. Surg. Oncol. 21:1647–1652. 24385210 10.1245/s10434-013-3467-7

[28] Emmertsen, K. 1985 Medullary thyroid carcinoma and calcitonin. Dan. Med. Bull. 32:1–28. 2859145

[29] Patlolla, J. M. , and C. V. Rao . 2015 Anti‐inflammatory and Anti‐cancer Properties of *β*‐Escin, a Triterpene Saponin. Curr. Pharmacol. Rep. 1:170–178.

[30] Guarino, V. , M. D. Castellone , E. Avilla , and R. M. Melillo . 2010 Thyroid cancer and inflammation. Mol. Cell. Endocrinol. 321:94–102. 19835928 10.1016/j.mce.2009.10.003

[31] Tan, S. M.‐L. , F. Li , P. Rajendran , A. P. Kumar , K. M. Hui , and G. Sethi . 2010 Identification of *β*‐escin as a novel inhibitor of signal transducer and activator of transcription 3/Janus‐activated kinase 2 signaling pathway that suppresses proliferation and induces apoptosis in human hepatocellular carcinoma cells. J. Pharmacol. Exp. Ther. 334:285–293. 20378717 10.1124/jpet.110.165498

[32] Wang, Y.‐W. , S.‐J. Wang , Y.‐N. Zhou , S.‐H. Pan , and B. Sun . 2012 Escin augments the efficacy of gemcitabine through down‐regulation of nuclear factor‐*κ*B and nuclear factor‐*κ*B‐regulated gene products in pancreatic cancer both in vitro and in vivo. J. Cancer Res. Clin. Oncol. 138:785–797. 22270965 10.1007/s00432-012-1152-z PMC11824426

[33] Patlolla, J. M. , J. Raju , M. V. Swamy , and C. V. Rao . 2006 *β*‐Escin inhibits colonic aberrant crypt foci formation in rats and regulates the cell cycle growth by inducing p21waf1/cip1 in colon cancer cells. Mol. Cancer Ther. 5:1459–1466. 16818504 10.1158/1535-7163.MCT-05-0495

